# Supplementary material for: Complex‐centric proteome profiling by SEC‐SWATH‐MS
Source: Mol Syst Biol. 2019 Jan 14;15(1):e8438. doi: 10.15252/msb.20188438 (PMC6346213; doi:10.15252/msb.20188438)
Supplement: Supplementary file 8 — Dataset EV7 [file MSB-15-e8438-s008.zip › feature_plots_string/P53384.pdf]

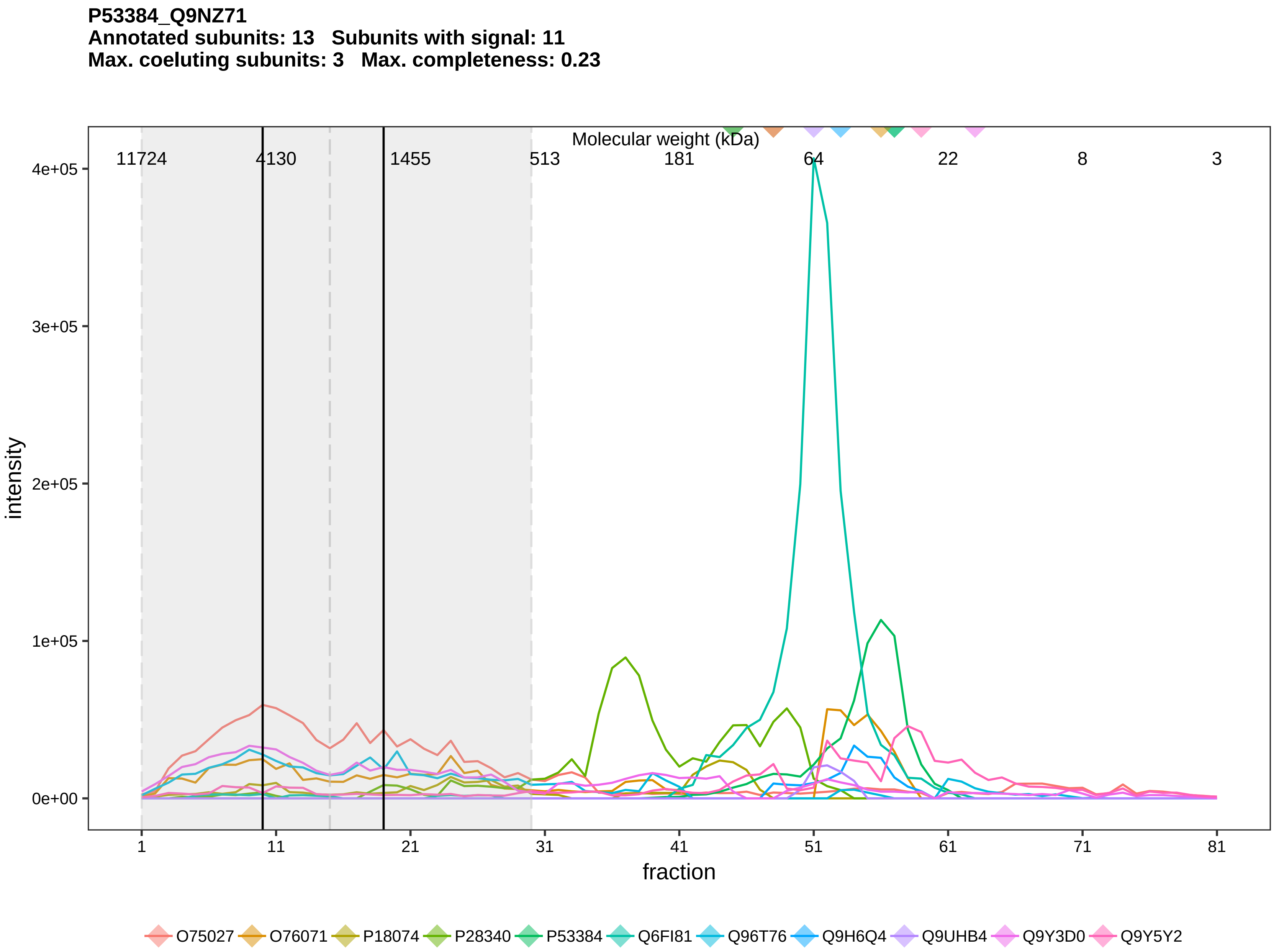
P53384_Q9NZ71
Annotated subunits: 13 Subunits with signal: 11
Max. coeluting subunits: 3 Max. completeness: 0.23
Molecular weight (kDa)
11724
4130
1455
513
181
64
22
8
3
intensity
4e+05
3e+05
2e+05
1e+05
0e+00
fraction
1
11
21
31
41
51
61
71
81
O75027
O76071
P18074
P28340
P53384
Q6FI81
Q96T76
Q9H6Q4
Q9UHB4
Q9Y3D0
Q9Y5Y2
